# Supplementary material for: Subcellular connectomic analyses of energy networks in striated muscle
Source: Nat Commun. 2018 Nov 30;9:5111. doi: 10.1038/s41467-018-07676-y (PMC6269443; doi:10.1038/s41467-018-07676-y)
Supplement: Supplementary file 7 — Supplementary Information [file 41467_2018_7676_MOESM7_ESM.pdf]

Supplementary Information

Subcellular Connectomic Analyses of Energy Networks in Striated Muscle

Bleck et al.

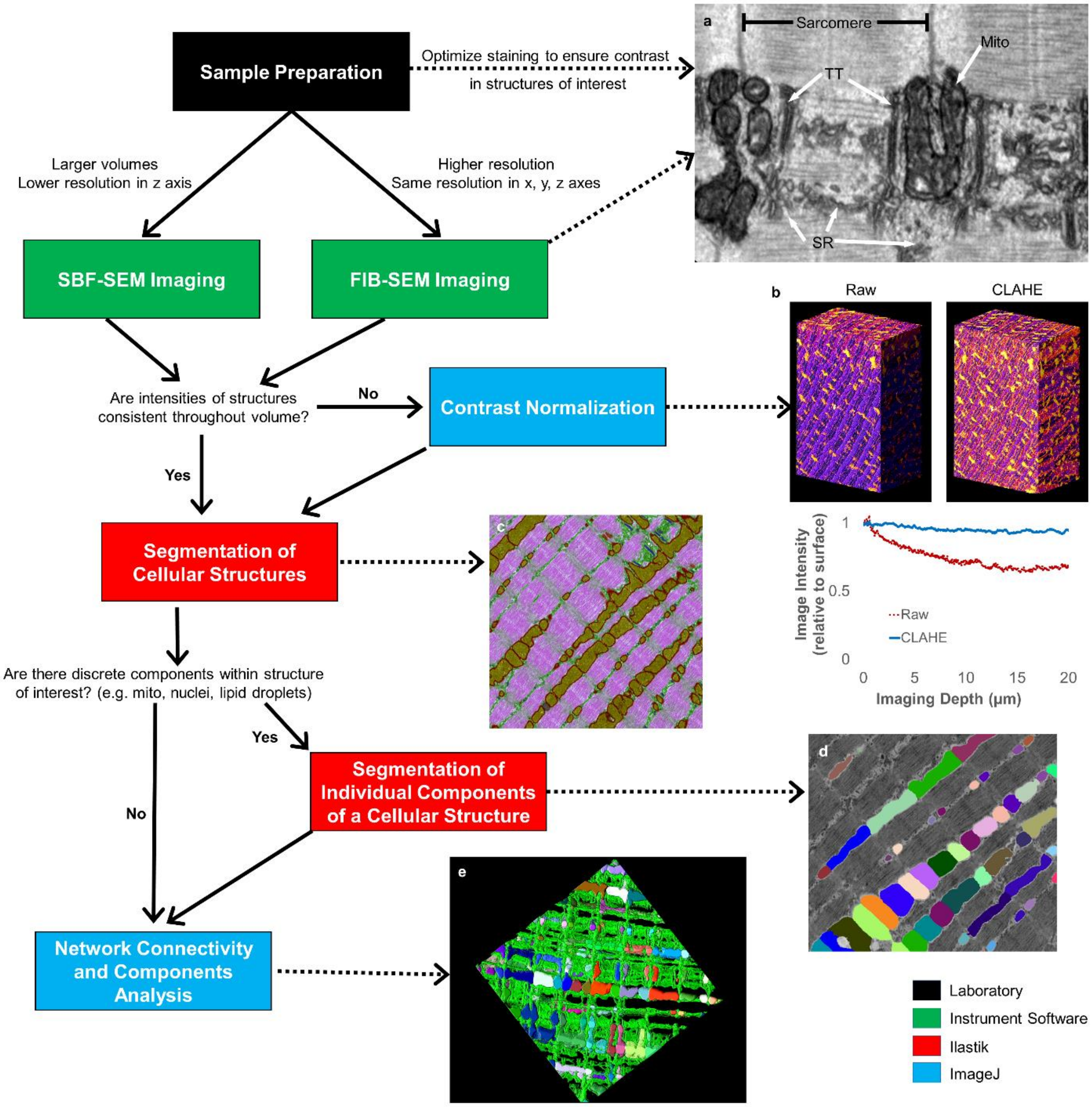

Supplementary Figure 1: Subcellular Connectomics Workflow. Muscle samples were prepared for SBF-SEM and FIB-SEM to maximize contrast in cellular membranes (a). Inconsistencies in contrast within cellular volumes are common with either imaging technique and were corrected using a standard ImageJ plugin (CLAHE) when necessary (b). Automated segmentation of cellular structures (c) and followed by segmentation of individual mitochondria (d) was performed using freely available Ilastik software. Analysis of mitochondrial networks, individual mitochondria within the networks, the junctions connecting mitochondrial networks, and mitochondrial interactions with other cellular structures (e – mitochondria/sarcoplasmic reticulum interactions) were performed using freely available tools and plugins for ImageJ.

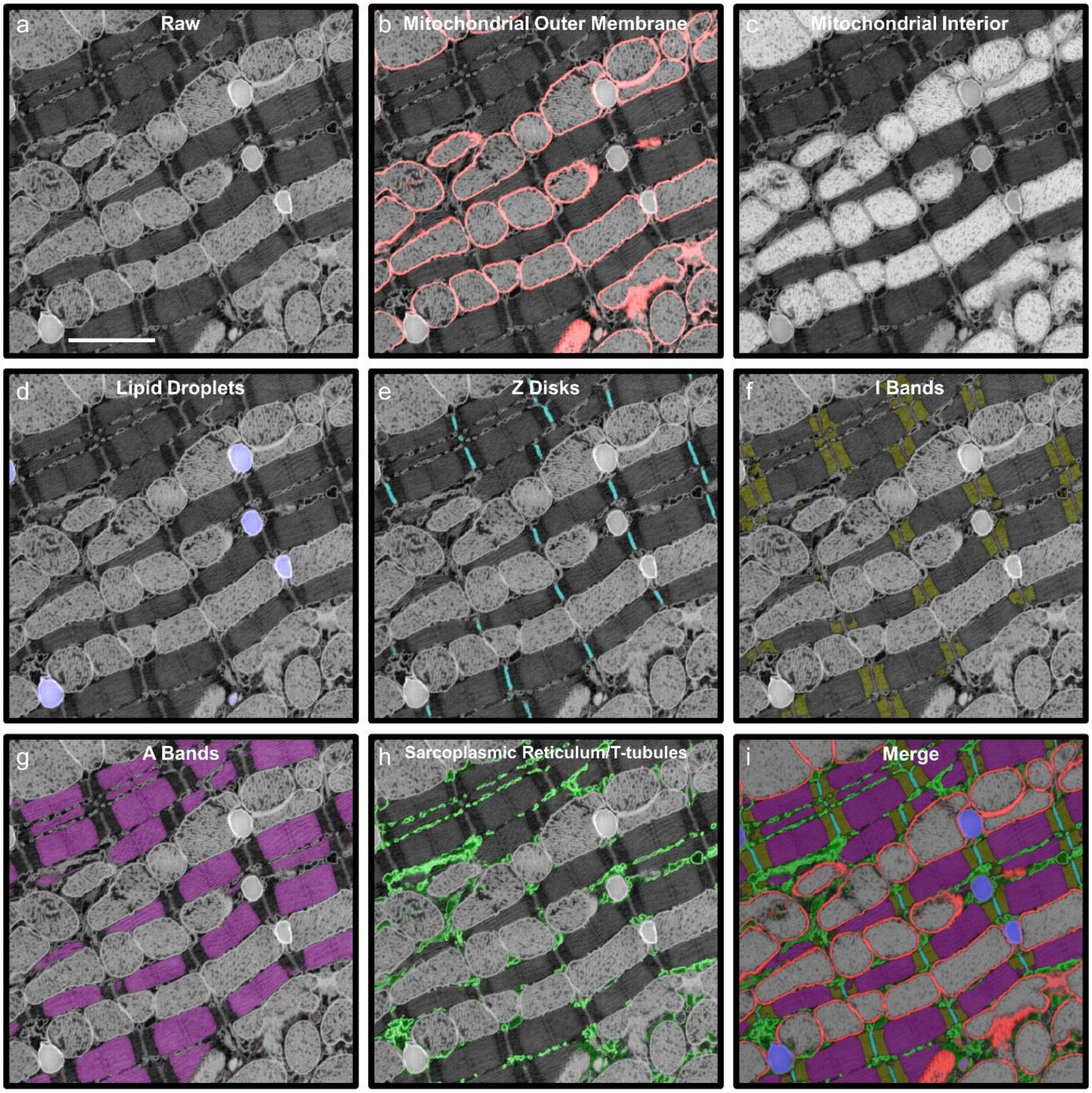

Supplementary Figure 2: Initial Segmentation of Cellular Structures. a) Representative single raw data image from a cardiac muscle FIB-SEM volume. Scale bar – 2 µm. b) Segmentation of mitochondrial outer membrane (red) overlaid on raw data. c) Segmentation of mitochondrial interior (white). d) Segmentation of lipid droplets (blue). e) Segmentation of sarcomeric z-disks (cyan). f) Segmentation of sarcomeric I-bands (yellow). g) Segmentation of sarcomeric A bands (magenta). h) Segmentation of sarcoplasmic reticulum and t-tubules (green). i) Combination of all segmented structures. Scale bar represents 2 µm.

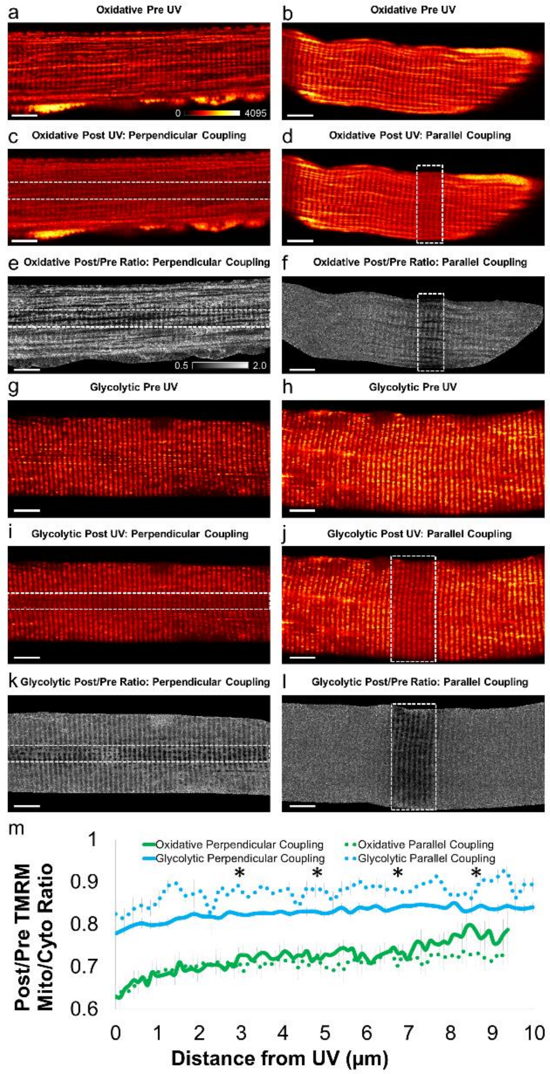

Supplementary Figure 3: Directional Analysis of Functional Mitochondrial Connectivity. a,b) TMRM and MitoPhotoDNP (MPD) loaded oxidative muscle fibers before UV irradiation. c,d) Oxidative fiber after horizontal and vertical MPD photoactivation, respectively. e,f) Oxidative fiber post/pre UV TMRM ratio images where dark pixels represent lower signal after UV and bright pixels represent increased signal. Images representative of 7 and 10 experiments, respectively from 3 mice. g,h) TMRM and MitoPhotoDNP (MPD) loaded glycolytic muscle fibers before UV irradiation. i,j) Glycolytic fiber after horizontal and vertical MPD photoactivation, respectively. k,i) Glycolytic fiber post/pre UV TMRM ratio images. Images representative of 13 and 10 experiments, respectively from 3 mice. m) Post/pre mitochondrial/cytosolic TMRM signal ratio as a function of distance away from the photoactivated region (white dotted lines). Data are means±SE. Mito/cyto TMRM ratio is proportional to mitochondrial membrane potential. Thus, a lower mito/cyto ratio suggests greater depolarization. Scale bar represents 10 µm. *denotes significant difference between glycolytic parallel and glycolytic perpendicular coupling.

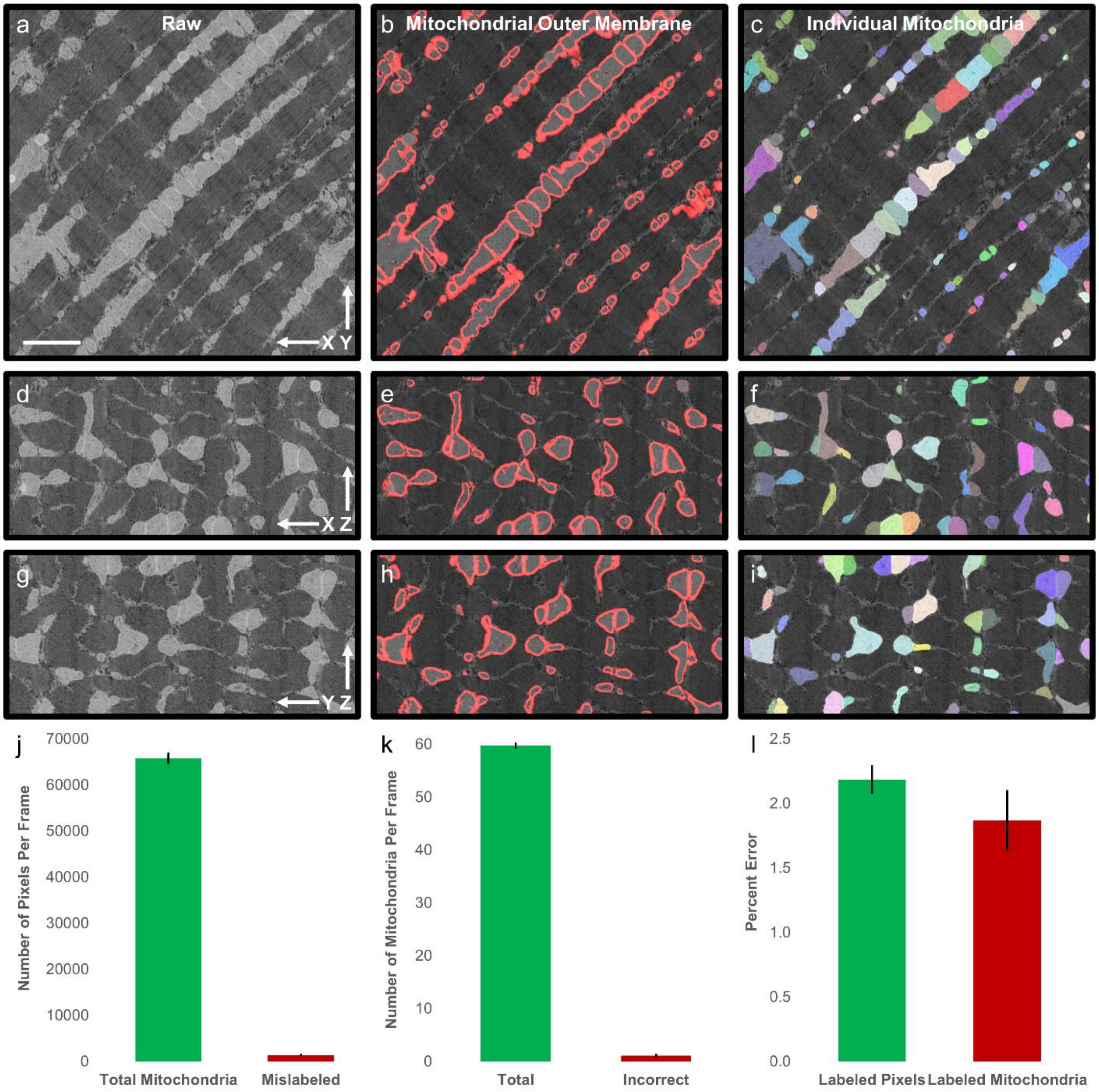

Supplementary Figure 4: Segmentation of Individual Mitochondria. a) Raw single XY image from an oxidative muscle FIB-SEM volume. Scale bar – 2 µm. b) Raw XY image overlaid with mitochondrial outer membrane segmentation (red) results. c) Raw XY image overlaid with individual mitochondrial segmentation results. Each color represents an individual mitochondrion. d-i) Corresponding XZ and YZ images for a-c. j) Total number of mitochondrial pixels and mislabeled pixels per frame. k) Total number of individual mitochondria and incorrectly labeled mitochondria per frame. l) Percent error of labeled pixels and mitochondria. Scale bar represents 2 µm.

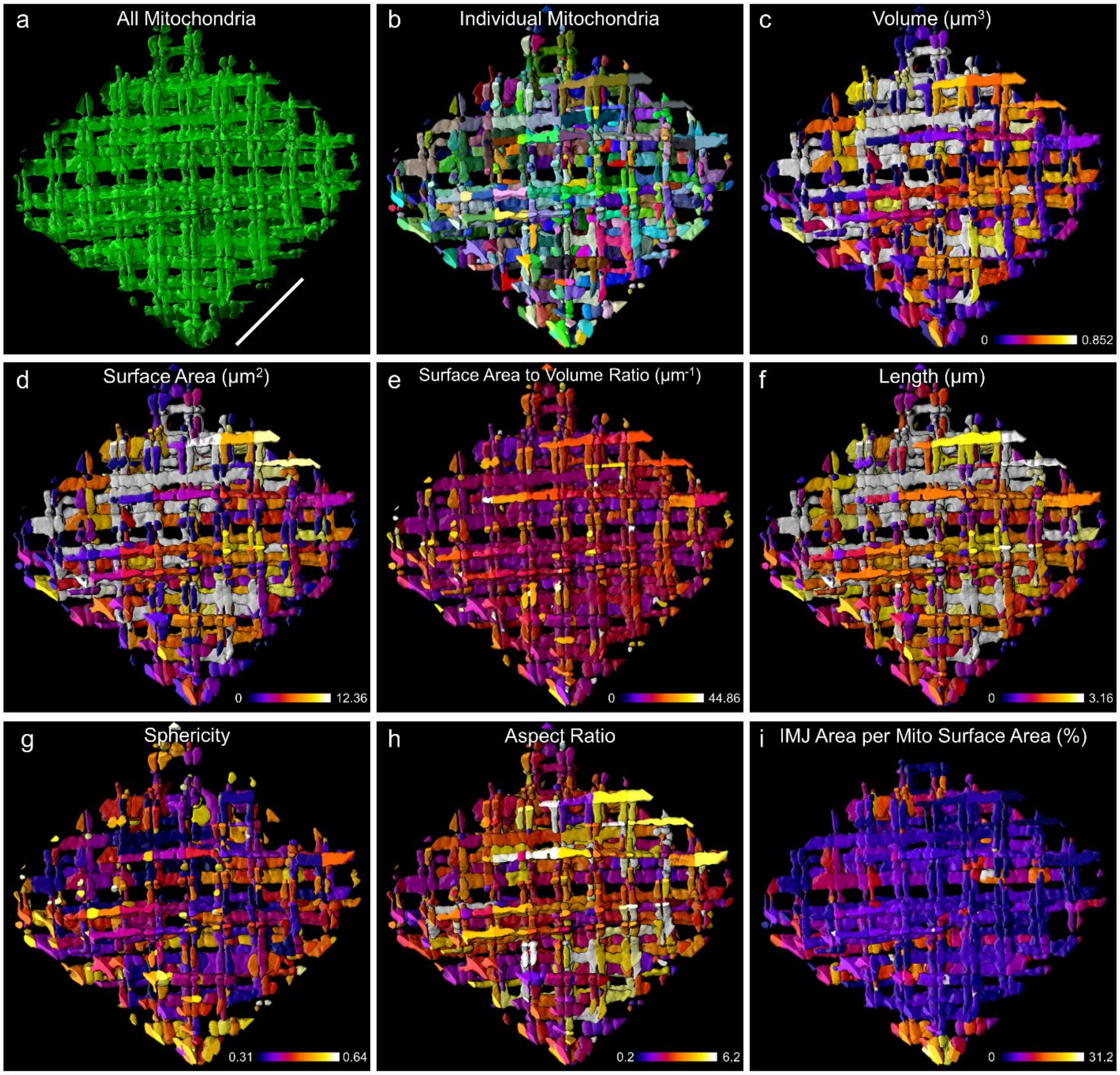

Supplementary Figure 5: 3D visualization of individual mitochondrial morphological characteristics. a) 3D rendering of all oxidative muscle mitochondria. b) Oxidative muscle individual mitochondria. Each color represents an individual mitochondrion. c) Mitochondria colored according to their volume. d) Mitochondrial surface area. e) Surface area-to-volume ratio f) Length g) Sphericity h) Aspect ratio. i) Percentage of intermitochondrial junction area per mitochondrial surface area. All look up tables scaled to the mean ± 2 standard deviations. Scale bar represents 5 μm.
