# Supplementary material for: Subcellular connectomic analyses of energy networks in striated muscle
Source: Nat Commun. 2018 Nov 30;9:5111. doi: 10.1038/s41467-018-07676-y (PMC6269443; doi:10.1038/s41467-018-07676-y)
Supplement: Supplementary file 8 — Description of Additional Supplementary Files [file 41467_2018_7676_MOESM8_ESM.docx]

**Legends for Supplementary Movies**

**Supplementary Movie 1:** Raw image stack from FIB-SEM volumes of cardiac (upper left), oxidative (upper right), and glycolytic (bottom) muscles followed by segmentation of cellular structures overlaid on the raw data. Movies have been down sampled to keep file size to a minimum.

**Supplementary Movie 2:** 360° rotation of a 3D rendering of individual mitochondria from a glycolytic (left), oxidative (middle), and cardiac (right) muscle FIB-SEM volumes.

**Supplementary Movie 3:** A segmented image stack (left) and 3D rendering (right) showing the tight coupling between donut mitochondria (cyan) and the sarcoplasmic reticulum (magenta) in glycolytic muscle.

**Supplementary Movie 4:** A segmented image stack (left) and 3D rendering (right) showing the tight coupling between donut mitochondria (cyan) and a lipid droplet (yellow) in cardiac muscle.

**Supplementary Movie 5:** 360° rotation of a 3D rendering of an oxidative muscle mitochondrial network (various colors represent individual mitochondria) and its interactions with the sarcoplasmic reticulum (green).

**Supplementary Movie 6:** 360° rotation of all lipid droplet connected mitochondria from an oxidative muscle FIB-SEM volume followed by a 360° rotation of all non-lipid droplet connected mitochondria. Lipid droplets – yellow, individual mitochondria – all other colors.
